# When is a high fat diet not a high fat diet?

**DOI:** 10.1186/1743-7075-2-27

**Published:** 2005-10-17

**Authors:** Richard D Feinman

**Affiliations:** 1 Department of Biochemistry, State University of New York Downstate Medical Center, Brooklyn, NY 11203 USA

## 

The observation that a high fat/low carbohydrate (CHO) diet has a beneficial effect on a mouse model of Alzheimer's disease (AD) published today is notable given previous results showing that high fat diets have a deleterious effect on AD. Van de Auwera, *et al*. [1] reported that mice fed a ketogenic diet (<1% carbohydrate, 80% fat) were found to have a 25% decrease in the protein Aβ42 compared to mice fed a standard high-carbohydrate, low-fat chow diet. Aβ42 is a particularly amyloidogenic mutant form of the amyloid precursor protein whose proteolytic product β-amyloid peptide is contained in the plaques and neurofibrillar tangles that are characteristic of AD.

Any suggestion of an environmental or dietary attack on AD is welcome given the devastating effects of the disease. Beyond the potential application, however, Van de Auwera, *et al*.'s results have general implications for nutritional approaches in biochemistry and cell biology, and ultimately on disease processes. The physiologic effect of dietary fat can be significantly modulated by the presence of carbohydrate and the associated hormonal changes. The description "high fat diet" is thus an inadequate way to characterize a diet. One must also specify the level of carbohydrate.

The principle that dietary fat might play a relatively passive role in metabolism and that the disposition of fat is regulated by the hormonal state stimulated by carbohydrate is taught in elementary courses in biochemistry but remains an under-appreciated factor in many studies, possibly due to the emphasis on low-fat recommendations of nutritional agencies. Because of the requirement of brain cells for glucose (or ketones) for energy metabolism and, in particular, because of the known involvement of insulin in regulating secretase (proteolytic enzyme in β-amyloid production) (e.g. [2]), it is pertinent to inquire about the role of macronutrient composition in the diet in neuronal disorders. The role of energy metabolism in brain function has been discussed in a recent review and hypothesis by Mukherjee and Seyfried [3].

In explaining the importance of macronutrient composition to students we emphasize energy metabolism and gain or loss of body weight and we stress the need to get away from the principle that "you are what you eat," and replace it with the idea that "you are what you do with what you eat [4]." A common analogy, that fat is the bomb and carbohydrate is the fuse, or in its original description, powder keg and tinder box, may be too broad for appreciation of fine control of metabolism but is probably good enough to illustrate the principle here. Although there are many effects of dietary change, to a first order approximation, carbohydrate is the major stimulus for insulin secretion and as an anabolic hormone, leads to repression of lipolysis and glycogenolysis. Continued hyperinsulinemia, therefore, may predispose to a state where dietary fat is stored rather than oxidized. In addition, current thinking on insulin resistance emphasizes the role of free fatty acids and other fat metabolites (e.g. [5,6]). One theory of the etiology of insulin resistance is that insulin resistance in the adipocyte represents down regulation of response due to continued hyperinsulinemia. This causes increased lipolysis and excessive liberation of fatty acids which may have several effects in peripheral tissues. Thus, the regulation of the TAG-fatty acid axis may be more important than the dietary levels of fat itself.

## Similarity of starvation and carbohydrate restriction

One of the areas bearing on the idea that disposition of fat is controlled by insulin is the observation made by several groups that the metabolic response to starvation resembles the response to carbohydrate restriction [7-11]. An important but, in our view, under-appreciated study is that of Klein & Wolfe [11] who compared responses of subjects on an 84 hour fast to the same subjects on a similar fast in which lipids were infused at a level equal to resting energy requirements. Table 1 shows that the levels of fatty acid, rates of oxidation and levels of glucose and ketone bodies were similar in the two groups (Table 1). These rather dramatic results were summarized by the authors as demonstrating that "carbohydrate restriction, not the presence of a negative energy balance, is responsible for initiating the metabolic response to fasting." It might be said that this is the key experiment in understanding the interaction between fat and carbohydrate. Similarly, Bisschop, et al. [8] demonstrated a similarity between high carbohydrate, low fat diets (CHO:Lipid:Protein = 85:0:15) and control (44:41:15) diets in FFA rate of appearance and oxidation but significant differences with a low carbohydrate, high fat diet (CHO:Lipid:Protein = 2:83:15).

**Table 1:** Similarity of starvation and carbohydrate restriction

**Reference [11]**	FFA (μmol/l)	fat oxidation (μmol/kg/min)	Glucose (mg/dl)	β-hydroxybutyrate (mM)
**Fast 84 h**	0.92	1.94	68	2.56
**Fast + Lipid**	1.02	1.67	66	2.54

Data from reference [11]. Procedure as described in the text.

## High fat diets in obesity

The interaction of fat and carbohydrate bear on the mechanism of weight loss strategies based on carbohydrate restriction. In considering the problem, it is important to recognize that percentages are misleading. There are really three degrees of freedom in design or analysis of a weight loss experiment: two of the three macronutrients and the total caloric intake. It is unlikely that the percentage rather than the absolute amount of macronutrients is the controlling variable and at least three published studies show that carbohydrate reduction is not necessarily accompanied by replacement with either fat or protein but rather caloric reduction due to the carbohydrate removed [12-14]. Such diets are effectively high fat by percentages but lead to substantial weight loss.

To understand the relative impact of carbohydrate and fat, one has to consider experiments in which one macronutrient is replaced by another. Several studies in the literature have demonstrated that in isocaloric comparisons, the replacement of dietary carbohydrate with fat leads to greater weight loss [15-19], that is, the absence of the fuse prevents an explosive effect of dietary fat on body mass. The limitations of the fat bomb *per se *are reviewed by Willett and Leibel [20].

## Cardiovascular disease

Many of the objections over the years of controlling the effect of dietary fat by replacing dietary carbohydrate has centered on the "obvious" danger that high fat diets present to plasma lipids and coronary risk. The subject is more complicated than obesity in that the mechanistic role of insulin is less well understood and the type of fat becomes much more important. Moreover, the relative importance of different risk factors LDL vs. triglycerides and HDL may be a matter of clinical judgment. However, what we know from epidemiology is that, whereas replacing unsaturated fat (UF) with saturated fat is deleterious to CVD outcome, replacing UF with carbohydrate is worse [21]. Similar effects are seen on the effect of dietary replacement on total cholesterol/HDL [22]. The generally beneficial effects of carbohydrate reduction on lipid profile has been reviewed by Volek [23].

## Diabetes

Control of lipid metabolism by insulin is of greatest importance in diabetes where insulin insufficiency or resistance is the key variable. The subject is a matter of current controversy since official organizations counsel against high-fat diets: the American Diabetes Association position statement, for example, presents an expert consensus for 60–70% of energy intake as carbohydrates for diabetics. Such high intake of the major insulin secretagogue as a therapeutic strategy remains counter-intuitive and may not even be known to or implemented by many practicing physicians and the idea has recently been strongly criticized [24,25].

A widely quoted example of experiments bearing on this question is the study of Garg, et al. [26,27]. In a four-center randomized crossover trial, carbohydrate was replaced with monounsaturated fats (CHO: 55% → 40; total fat: 30% → 45) or, conversely, MUF was replaced with CHO. It was found that replacement of MUF with carbohydrate "caused persistent deterioration of glycemic control and accentuation of hyperinsulinemia, as well as increased plasma triglyceride and very-low-density lipoprotein cholesterol levels, which may not be desirable [26]," that is, the high fat arm was beneficial, again, suggesting that what happened to the fat was more important than its concentration in the diet.

**Figure 1:**
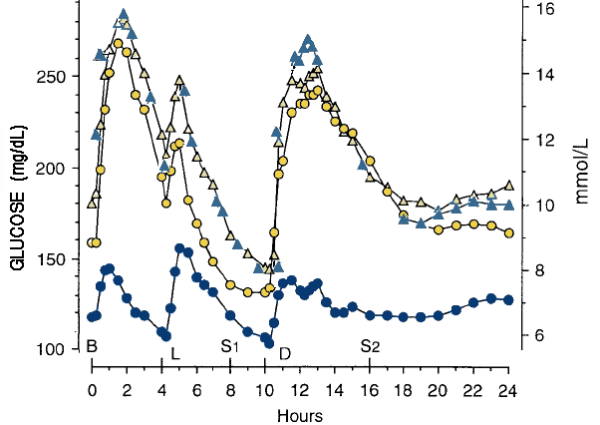
**Effect of diet on glucose**. Mean plasma glucose concentration before (triangles) and after 5 weeks on control diet (yellow circles: (CHO:fat:protein = 55:30:15)) or 5 weeks on the higher fat diet (blue circles: (20:50:30)). Meals are Breakfast (B), lunch (L) and dinner(D) plus 2 snacks (S1, S2). Data from reference [28].

To determine the relative importance of fat and carbohydrate and protein, Gannon and coworkers have measured the effect of replacement of carbohydrate with protein and fat in patients with type 2 diabetes [28]. Figure 1 shows the effects on glycemic control of the replacement of carbohydrate with protein and fat. Again, the 50% fat diet provides better glycemic control although, in this case, the relative contributions of carbohydrate reduction and increase in protein is unknown.

## Conclusion

The long range implication of Van de Auwera's study for AD remains to be seen but the general lesson is that in dietary recommendations or in testing animal models a diet should not be characterized as high fat without also specifying the level of carbohydrate.
